# Establishing a low-risk zone for a temporary extra-articular calcaneo-tibial pin fixation in an unstable ankle or subtalar joint

**DOI:** 10.1038/s41598-022-17490-8

**Published:** 2022-08-03

**Authors:** Ik Yang, Ho Won Lee, Huiying Xu, Seung Rim Kang, Hyong Nyun Kim

**Affiliations:** 1 Department of Radiology, Kangnam Sacred Heart Hospital, Hallym University College of Medicine, Seoul, Republic of Korea; 2 Department of Orthopedic Surgery, Kangnam Sacred Heart Hospital, Hallym University College of Medicine, 948-1, Dalim-1dong, Youngdeungpo-gu, Seoul, 150-950 Republic of Korea; 3 Department of Ultrasound, The First Hospital of Jilin University, Changchun, Jilin China; 4 Department of Radiology, Soonchunhyang University Gumi Hospital, Gumi, Republic of Korea

**Keywords:** Anatomy, Health care

## Abstract

This study aimed to establish a low-risk zone to avoid neurovascular injury during a temporary extra-articular calcaneo-tibial pin fixation in an unstable ankle or subtalar joint. A line from the calcaneal tuberosity center to the lateral end of the posterior malleolus at the ankle joint level defines the lateral border of this zone. Another line from the calcaneal tuberosity center to the midpoint of the anterior distal tibial articular surface at the joint level defines its medial border. This region was assumed to have a low neurovascular injury risk upon pin insertion. Fifty ankles from 50 patients who had undergone magnetic resonance imaging (MRI) for ankle disorders were assessed. T1-weighted oblique axial MRI slices were oriented to the pin trajectory. The mean distances between the sural nerve and the lateral border of the low-risk zone and between the posterior tibial neurovascular structures and the medial border of the low-risk zone were 15.0 ± 2.5 (range 9.1 to 21.1) and 12.8 ± 2.6 (6.3 to 20.8) mm, respectively. No neurovascular structures were identified within the low-risk zone. These findings demonstrated that an unstable ankle or subtalar joint can be temporarily fixated with an extra-articular calcaneo-tibial pin at a defined zone with a low neurovascular injury risk.

## Introduction

**Figure 1:**
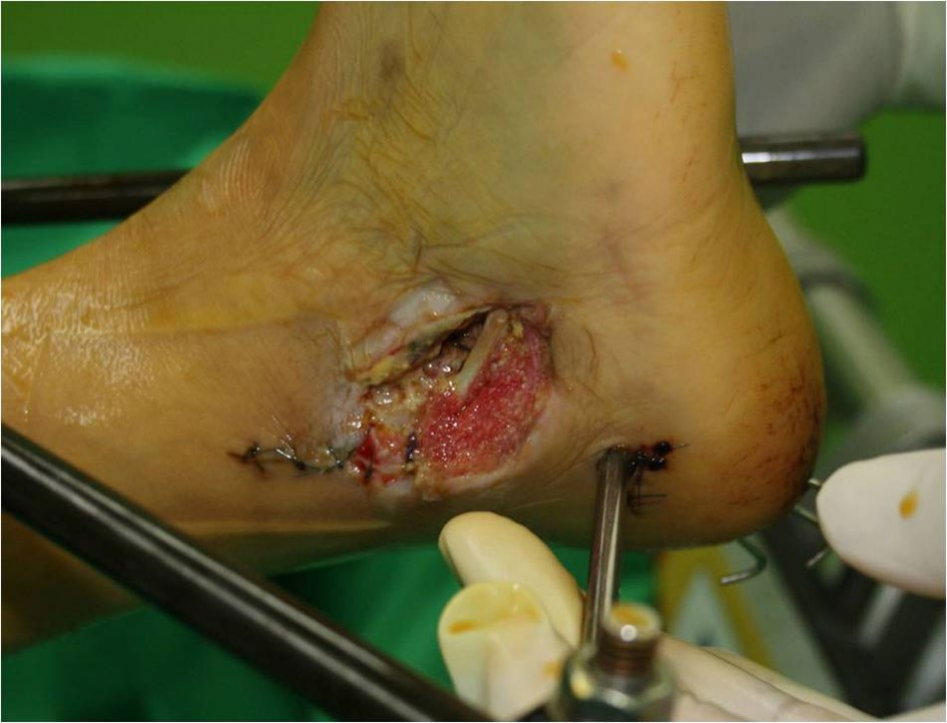
An external fixator with multiple pins and bars may theoretically increase the risk of pin-tract infections. When these pins are closely located to the wounds, it may be difficult to dress and to seal up the wound for a negative pressure therapy when indicated.

The use of temporizing external fixation prior to the definitive management of periarticular fracture associated with compromised soft tissue envelope is the established procedure for a high-energy trauma^1,2^. It can maintain alignment after fracture reduction, reduce the movement of the fractured area, and reduce the pain and the following inflammatory reactions of neighboring tissues, helping the swollen soft tissue to subside^3,4^. The benefit of staged operation with the use of the temporary external fixation includes decreased wound complications and infections^2,5,6^. An external fixator can also be quickly applied to fix an unstable ankle in critical polytrauma patients when time is an essential component of damage control^7–10^. Various types of external fixators have been developed, including multiple pins and bars constructs (delta frame) or a circular ring fixator^10^. However, to form a stable construct, multiple pins, bars, and wires are required, which may theoretically increase the risk of pin-tract infections. There are neurovascular injury risks during percutaneous insertion of the pins^4^. In a study of 52 distal tibial fractures which were temporarily fixated with bridging external fixators, medial calcaneal nerve was injured in 2 cases (4%)^4^. When these pins and wires are closely located to the wounds, it may be difficult to dress and seal up the wound for a negative pressure therapy when indicated (Fig. 1). Furthermore, construct costs are high.

The vertical trans-articular pin fixation was developed in the 1960s to maintain the joint alignment of an unstable ankle^11–13^. It is still used these days as it is an inexpensive, simple, and quick method for stabilizing the ankle joint^14^. A pin is inserted retrograde from the plantar side of the calcaneus passing through the subtalar joint and the talar body and up to the tibia to stabilize the ankle joint. However, drilling through a joint will damage the articular cartilage and can contribute to postinjury arthritis or the development of an iatrogenic cyst^15,16^. There is also the risk of neurovascular injury during pin insertion through the plantar side of the foot because the plantar nerve and vascular structures are located here. Furthermore, the pin can migrate upward into the tibial medullary canal, making it difficult to remove.

**Figure 2:**
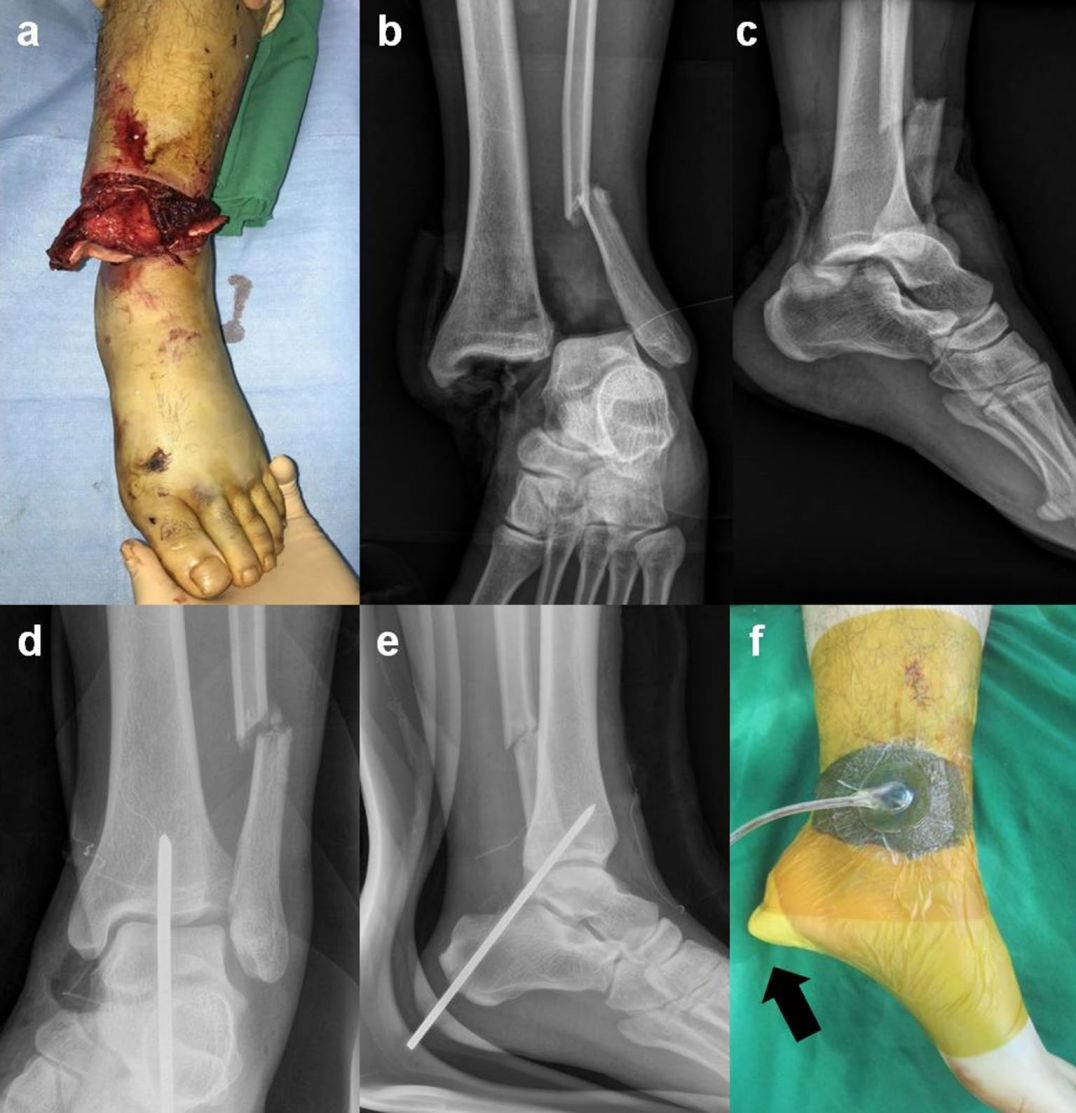
**(a,b,c)** A 27-year-old male patient presented with ankle dislocation with contaminated soft tissue damage. **(d,e)** A temporary extra-articular calcaneo-tibial pin fixation stabilized the dislocated or unstable ankle joint after reduction without injuring the articular cartilage of the ankle and the subtalar joints. **(f)**The wound could be sealed up for negative pressure therapy. The arrow indicates the pin covered with gauze to prevent it from penetrating the film.

A temporary extra-articular calcaneo-tibial pin fixation can stabilize the dislocated or unstable ankle joint after reduction without injuring the articular cartilage of the ankle and the subtalar joints (Fig. 2)^17–20^. However, it may not be indicated for a pilon fracture or more proximally based fractures. In a biomechanical cadaveric study, there was no significant difference in stiffness between the vertical trans-articular fixation and the extra-articular calcaneo-tibial fixation^20^. Compared to external fixators, the use of a single pin in a temporary extra-articular calcaneo-tibial fixation makes it much simpler and quicker to apply, and there are no bars or any constructs near the wound that limit its management^19^. Furthermore, the cost of a single pin is much cheaper. However, the pin must pass through the posterior extra-articular space of the ankle joint, putting the posterior tibial neurovascular structures and the sural nerve at risk.

**Figure 3:**
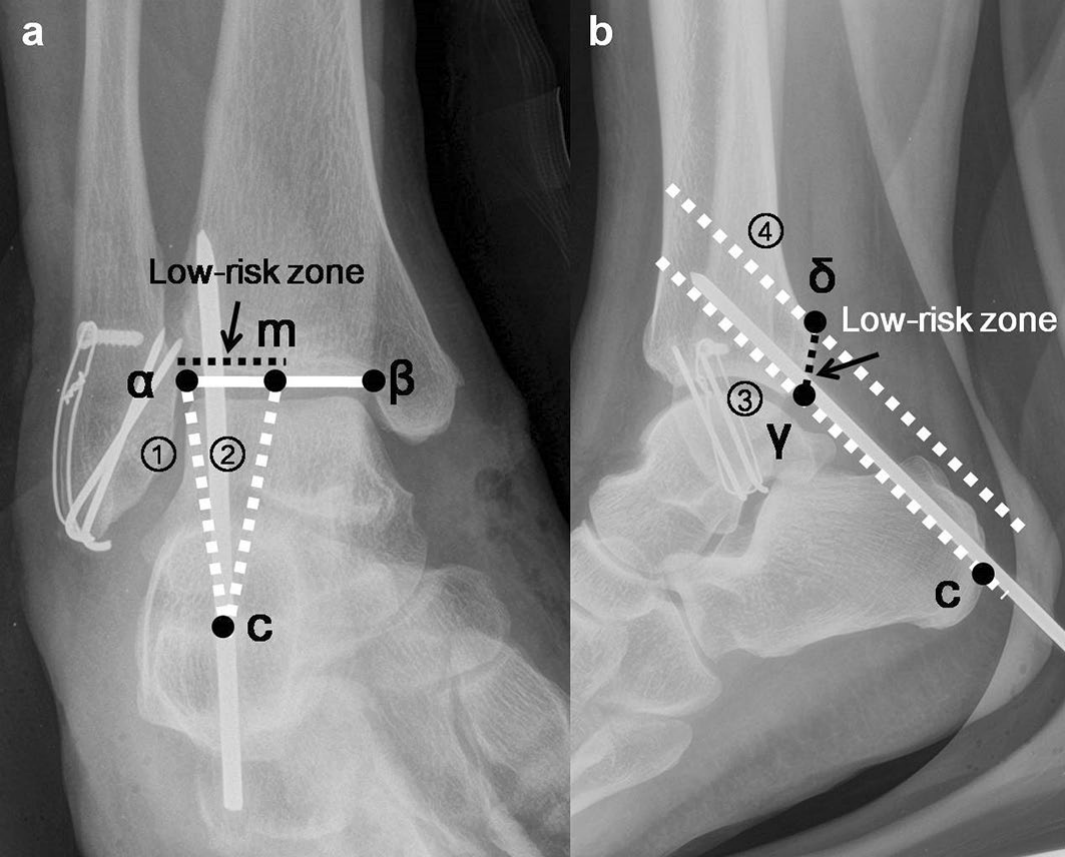
**(a)** We assumed that it will be safe to insert a pin from the center of the calcaneal tuberosity (c) to the distal tip of the posterior malleolus through the posterior extra-articular ankle space when it is inserted medial to the lateral end of the posterior malleolus (α) (lateral border of the low-risk zone:①) and lateral to the midpoint of the anterior distal tibial articular surface (m) (medial border of the low-risk zone:②) at the joint level on the C-arm fluoroscopic ankle mortise radiograph. **(b)** On the C-arm fluoroscopic lateral ankle radiograph, pin insertion from the center of the calcaneal tuberosity (c) to the distal tip of the posterior malleolus (γ) corresponded to the inferior border of the low-risk zone (③) and the line parallel to the inferior border of the low-risk zone and 1 cm proximal (δ) was the superior border of the low-risk zone (④). The arrows indicate the low-risk zone.

The posterior extra-articular ankle space is commonly approached during two-portal posterior ankle arthroscopy. Through many cadaveric and MRI studies, it is known that when the arthroscopic instruments stay lateral to the flexor hallucis longus tendon, the posterior tibial neurovascular structures are safe^21–23^. However, for calcaneo-tibial pin fixation, the flexor hallucis longus tendon cannot be used as a landmark. Instead, we used C-arm fluoroscopic ankle mortise and lateral radiographs to establish a low-risk zone on the posterior extra-articular space to avoid neurovascular injury during pin fixation. We assumed that it will be safe to insert a pin from the center of the calcaneal tuberosity to the distal tip of the posterior malleolus through the posterior extra-articular ankle space when it is inserted medial to the lateral end of the posterior malleolus (lateral border of the low-risk zone) and lateral to the midpoint of the anterior distal tibial articular surface (medial border of the low-risk zone) at the joint level on the C-arm fluoroscopic ankle mortise radiograph (Fig. 3) and within 1 cm proximally from the distal tip of the posterior malleolus on the lateral radiograph.

The purpose of the current study was to measure the distance between the sural nerve and the lateral border of the low-risk zone and the distance between the posterior tibial neurovascular structures and the medial border of the low-risk zone on the MRI to verify the safety of extra-articular calcaneo-tibial pin fixation.

## Methods

### Study participants

Fifty ankles from 50 patients (30 males, 20 females) with a mean age of 45.6 years (range 16 to 79 years) who had undergone magnetic resonance imaging (MRI) for ankle disorders between November 2020 and June 2021 were assessed. The institutional review board approval was obtained [IRB of Hallym University Kangnam Sacred Heart Hospital (IRB-2017–12-004)]. All methods were performed in accordance with the relevant guidelines and regulations. The study focused on MRIs taken in the normal clinical practice for ankle disorders, and the informed consent from the patients was waived by the IRB of Hallym University Kangnam Sacred Heart Hospital. The MRIs were taken to evaluate for chronic ankle problems, including chronic ankle instability, osteochondral lesion of the talus, chronic ankle pain, etc. We excluded the patients aged less than 16 years because extra-articular calcaneo-tibial pin stabilization is not indicated for distal tibia and calcaneus with open growth plates. Patients with ankle joint deformity, such as varus ankle arthritis, or with conditions that can distort normal anatomy were excluded from the measurement. Patients with previous ankle surgeries or with metal hardware were also excluded.

### Description of the operative technique

**Figure 4:**
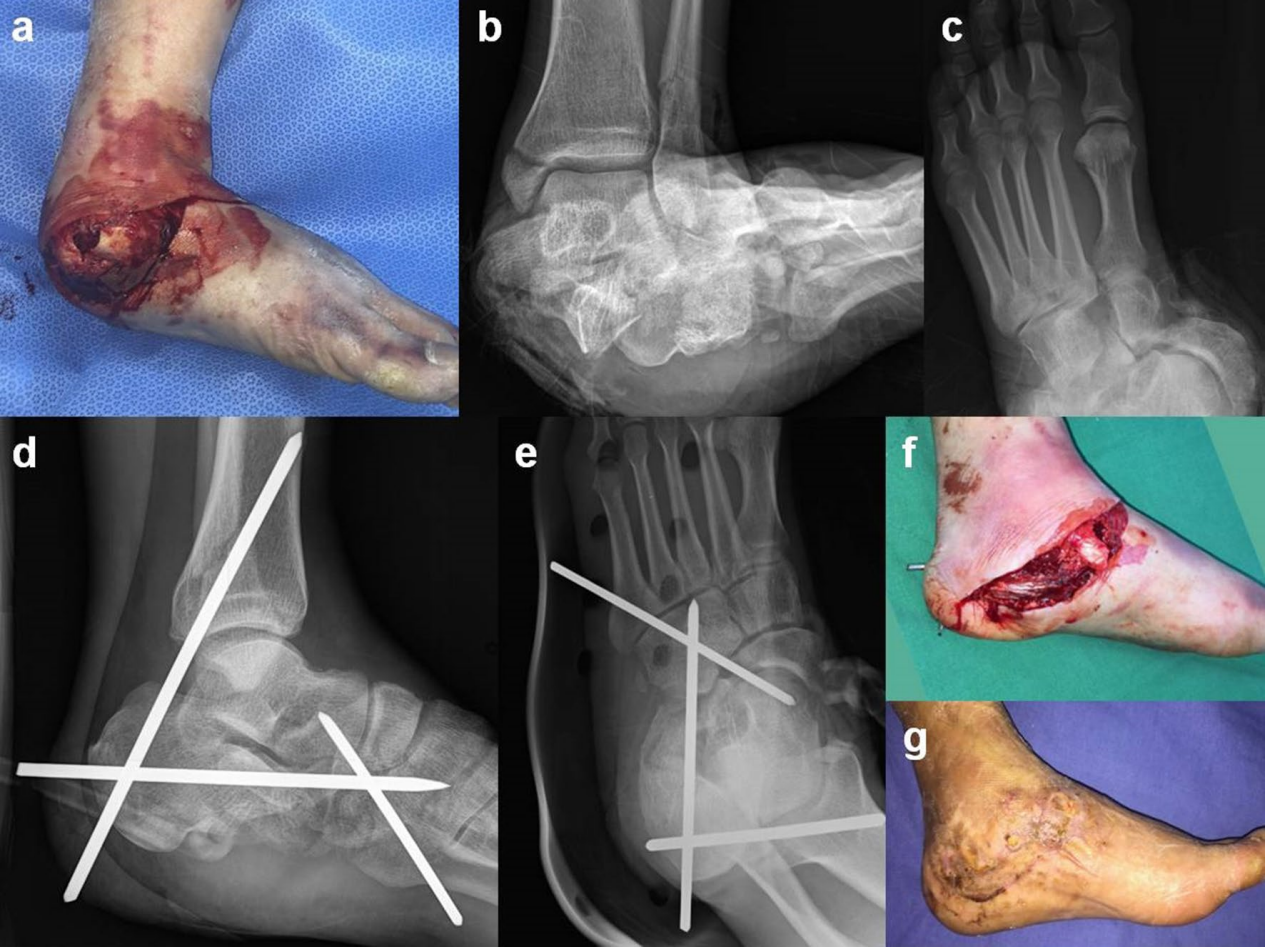
**(a,b,c)** A 56-year old male patient sustained subtalar and mid-tarsal dislocation with tibial neurovascular injury. **(d,e)** Temporary extra-articular calcaneo-tibial pin fixation stabilized the unstable subtalar joint, which then enabled repair of neurovascular structures. **(f)** Compared to a circular ring fixator or multiple pins and bars constructs, calcaneo-tibial pin fixation enables faster stabilization and a better view and working space for neurovascular repair and wound care. **(g)**The wound healed with a skin graft, while the fracture healed with delayed firm internal fixation.

The temporary extra-articular calcaneo-tibial pin stabilization can be performed in a supine, lateral, or prone position depending on the requirements of concomitant procedures. In the supine position, the hip is externally rotated, and the knee is flexed to make a Fig. 4 position, exposing the posterior heel from the operating table. With the ankle in the neutral position, a large diameter non-threaded Steinman pin is inserted at the center of the calcaneal tuberosity inferior to the most prominent posterior point. The pin is then advanced to the distal tip of the posterior malleolus within 1 cm proximally. The C-arm fluoroscopic ankle mortise radiograph is used during the insertion of the pin to check if it stays medial to the lateral end of the posterior malleolus and lateral to the midpoint of the anterior distal tibial articular surface at the joint level. The distal end of the pin is then cut 2–3 cm from the heel for later removal. The pin should not penetrate the anterior tibial cortex because when it is advanced proximally, it can injure the anterior neurovascular structures and embed the distal end of the pin into the skin, making it difficult to remove (Fig. 4). After the pin fixation, the heel should be well padded, and the ankle can be further supported with a short leg splint.

### MRI assessment

**Figure 5:**
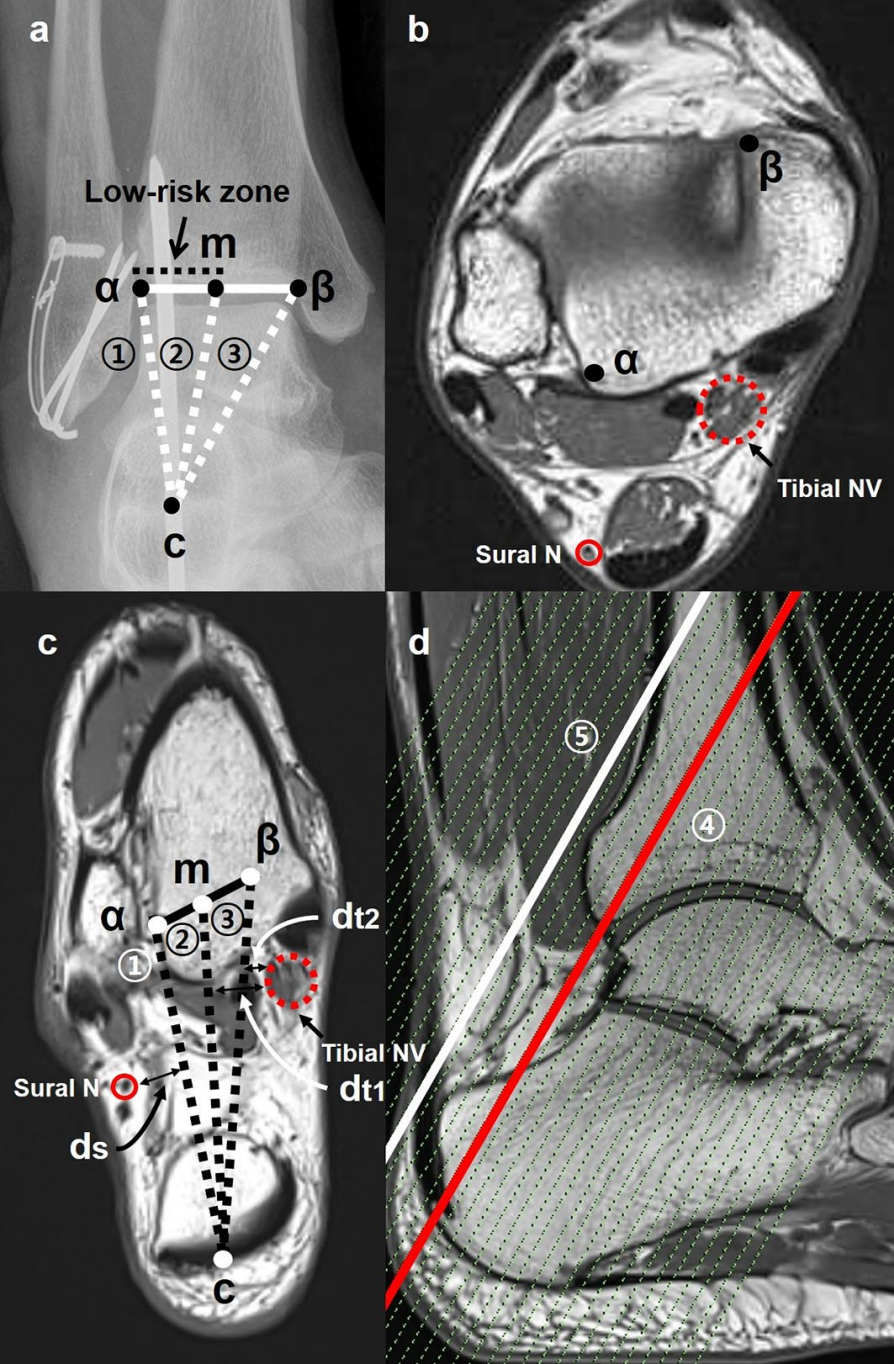
**(a)** The C-arm fluoroscopic ankle mortise radiograph is used during pin insertion. The low-risk zone on the simple radiograph was transferred to the MRI to measure the distance between its borders and the neurovascular structures**. (b)** On the axial image, the lateral end of the posterior malleolus at the joint level (α) and the anteromedial end of the anterior distal tibial articular surface (β) are marked. The marks can be shown in every oblique axial slice using Pictured Archives and Communication System (PACS). **(c)**The oblique axial slice, oriented on the trajectory of the pin fixation (from the center of the calcaneal tuberosity to the distal tip of the posterior malleolus: ④), was selected for the measurement. A line was drawn connecting the two ends (α, β). Then, a line was drawn from the center of the calcaneal tuberosity (c) bisecting (m) the line connecting the two ends (α, β), indicating the medial border of the low-risk zone (②). Also, for the low-risk zone’s lateral border (①), a line was drawn from the center of the calcaneal tuberosity (c) to the lateral end of the posterior malleolus (α). Similarly, for the most medial border (③), a line was drawn from the center of the calcaneal tuberosity (c) to the anteromedial end of the anterior distal tibial articular surface (β), allowing us to confirm the safety of aiming the pin toward the medial end of the anterior distal tibial articular surface during fixation. The three borders of the low-risk zone are observable in every oblique slice using PACS. The distance (d_s_) between the sural nerve (closed circle) and the lateral border of the low-risk zone (①), (d_t1_) the posterior tibial neurovascular structures (dotted circle) and the medial border of the low-risk zone (②), and (d_t2_) the posterior tibial neurovascular structures and the most medial border of the low-risk zone (③) were measured. **(d)** Five additional consecutive oblique axial slices within 1 cm proximal from the distal tip of the posterior malleolus (④) were recorded to determine the safety of inserting the pin into the posterior malleolus 1 cm proximal from the distal tip (⑤).

A Siemens 3.0-T MR scanner (MAGNETOM Skyra, Siemens Healthcare, Erlangen, Germany) was used for the study, and all measurements were made from the Pictured Archives and Communication System (PACS) (Fig. 5). To measure the distance between the neurovascular structures and the borders of the low-risk zone, where the pin should be inserted, T1 oblique axial slices were oriented parallel to the trajectory of the pin fixation, which was from the center of the calcaneal tuberosity to the distal tip of the posterior malleolus (slice 1). This enabled the measurement of the closest distance between the neurovascular structures and the pin when it is inserted inside the low-risk zone (Fig. 5c).

T1 oblique axial slices were measured with a thickness of 2 mm. The center of the calcaneal tuberosity was marked where it was most prominent to the posterior side. This mark could be highlighted in every oblique axial slice using the PACS program. The lateral end of the posterior malleolus at the joint level and the anteromedial end of the anterior distal tibial articular surface were marked, and a line was then drawn to connect the two ends. Then, a line corresponding to the medial border of the low-risk zone was drawn from the center of the calcaneal tuberosity bisecting the line connecting the two ends. For the lateral border, a line was drawn from the center of the calcaneal tuberosity to the lateral end of the posterior malleolus at the joint level. Also, a line was drawn from the center of the calcaneal tuberosity to the anteromedial end of the anterior distal tibial articular surface. This line indicated the most medial border, allowing us to check if it is safe to aim the pin toward the medial end of the anterior distal tibial articular surface during fixation. The three borders of the low-risk zone could be shown in every oblique slice using the PACS program. The distance between the sural nerve and the lateral border of the low-risk zone was measured. Similarly, the distance between the posterior tibial neurovascular structures and the medial border of the low-risk zone and between the posterior tibial neurovascular structures and the most medial border were also measured. The sural nerve and the posterior tibial neurovascular structures could be best identified on the T1 axial image at the joint level and could be traced on the T1 oblique axial slices using the PACS program. Additionally, five consecutive oblique axial slices within 1 cm proximal from the distal tip of the posterior malleolus were conducted to determine the safety of inserting the pin into the posterior malleolus 1 cm proximal from the distal tip. The T1 oblique axial slice from the center of the calcaneal tuberosity to the distal tip of the posterior malleolus was the primary slice for the analysis and five more slices were added for the analysis for each of the 50 ankles. One musculoskeletal radiologist and one orthopedic surgeon, both with more than 10 years of experience, measured the distances twice with an interval of one week. The mean values with standard deviation, range and 95% confidence intervals were presented. The inter- and the intra-observer reliabilities of the measurement were assessed using the intraclass correlation coefficient (ICC) with a 95% confidence interval, calculated by a 2-way mixed model. The absolute agreement was measured. The ICC was interpreted as poor agreement (< 0.50), moderate agreement (0.50–0.75), good agreement (0.75–0.90), or excellent agreement (> 0.90)^24^. Statistical analysis was performed using the SPSS version 21.0 (IBM Corporation, Armonk, NY, USA).

### Ethical approval

This study was approved by IRB of Hallym University Kangnam Sacred Heart Hospital (IRB-2017–12-004).

## Results

The results are summarized in Table 1. The mean distance between the sural nerve and the lateral border of the low-risk zone was 15.0 ± 2.5 (range 9.1 to 21.1) mm, while the mean distance between the posterior tibial neurovascular structures and the medial border of the low-risk zone was 12.8 ± 2.6 (range 6.3 to 20.8) mm on the T1-weighted oblique axial MRI slice (slice 1), which included the trajectory of the pin fixation (from the center of the calcaneal tuberosity to the distal tip of the posterior malleolus). No neurovascular structures were identified in the low-risk zone among all the cases. The intra- and inter-observer reliability of the measurement of the distance between the borders of the low-risk zone and the neurovascular structures was acceptable (Table 2).

**Table 1:** Distance of neurovascular structures from the borders of the low-risk zone.

Slice number*	Low-risk zone						Tibial NV to most medial border		
	Sural N to lateral border			Tibial NV to medial border					
	Mean ± SD	Range		Mean ± SD	Range	95% CI	Mean ± SD	Range	95% CI
1	15.0 ± 2.5	9.1 − 21.1	14.7 − 15.6	12.8 ± 2.6	6.3 − 20.8	12.4 − 13.1	5.3 ± 2.2	0.5 – 12.4	5.0 – 5.6
2	13.9 ± 2.3	8.9 – 19.8	13.6 – 14.2	11.8 ± 2.6	5.7 – 19.9	11.5 – 12.2	4.1 ± 2.3	− 1.12 – 11.3	3.8 – 4.4
3	12.6 ± 2.5	8.0 – 19.2	12.3 – 13.0	11.0 ± 2.7	5.2 – 17.7	10.7 –11.4	2.8 ± 2.6	− 2.6 – 10.1	2.4 – 3.2
4	11.3 ± 2.5	6.4 – 17.2	11.0 – 11.7	10.2 ± 2.9	4.7 – 17.2	9.8 – 10.6	1.3 ± 2.9	− 4.7 – 8.2	0.9 – 1.7
5	10.0 ± 2.7	2.9 – 16.3	9.6 – 10.4	9.2 ± 3.0	2.4 – 16.4	8.8 – 9.7	− 2.2 ± 3.1	− 6.8 – 7.7	− 0.7 – 0.2
6	8.4 ± 2.8	2.4 – 15.4	8.0 – 8.8	8.1 ± 3.1	2.6 – 16.3	7.6 – 8.5	− 2.0 ± 3.3	− 8.4 – 6.7	− 2.0 to − 1.5

* T1 oblique axial slices were made with a thickness of 2 mm parallel to the trajectory of the pin fixation which was from the center of the calcaneal tuberosity to the to the distal tip of the posterior malleolus (slice 1). The values (mm) are presented as the mean with the standard deviation, range and 95% confidence interval. The negative ( −) values indicate that the neurovascular structures crossed the border. *N* nerve, *NV* neurovascular structure, *SD* standard deviation, *CI* confidence interval.

**Table 2:** Inter-observer and intra-observer reliability of MRI measurements.

	Low-risk zone				Tibial NV to most medial border	
	Sural N to lateral border		Tibial NV to medial border			
	ICC	95% CI	ICC	95% CI	ICC	95% CI
Inter-observer reliability	0.81	0.63 – 0.90	0.74	0.53 – 0.85	0.75	0.47– 0.87
Intra-observer reliability						
Observer 1	0.87	0.74 – 0.93	0.75	0.53 – 0.86	0.84	0.73 – 0.91
Observer 2	0.85	0.70 – 0.92	0.84	0.71 – 0.91	0.84	0.71 – 0.91

*N* Nerve*, NV* Neurovascular structure, *ICC* Intraclass correlation coefficient, *CI* Confidence interval.

The distance between the neurovascular structures and the borders of the low-risk zone decreased on the proximal slices. The shortest mean distance from the sural nerve to the lateral border (8.4 ± 2.8 [range, 2.4 to 15.4] mm) and from the posterior neurovascular structures to the medial border (8.1 ± 3.1 [range, 2.6 to 16.3] mm) was on the most proximal slice (slice 6). There were no neurovascular structures located inside the low-risk zone within 1 cm proximal of the distal tip in all the cases. The mean distance between the posterior tibial neurovascular structures and the most medial border of the low-risk zone was 5.3 ± 2.2 (range, 0.5 to 12.4) mm on the slice 1. However, in the proximal slices (from slice 2 to 6), there were cases wherein the posterior tibial neurovascular structures were located lateral to the most medial border (slice 2: 4%, slice 3: 18%, slice 4: 34%, slice 5: 52%, slice 6: 71%), placing them at risk of being damaged when the pin is inserted along the most medial border toward the medial end of the anterior distal tibial articular surface. On the most proximal slice (slice 6), the mean distance between the posterior tibial neurovascular structures and the most medial border of the low-risk zone was − 2.0 ± 3.3 (range − 8.4 to 6.7) mm.

## Discussion

The percutaneous placement of wires, pins, or screws can sometimes cause iatrogenic neurovascular injury, especially when it is performed through the thick layers of soft tissues^4^. Iatrogenic damage from external fixator pins has been reported to result in pseudoaneurysms and other clinical complications, such as partial or complete occlusion of arteries or persistent neurologic symptoms^25^. As such, it is essential to understand the anatomy of the neurovascular structures around the sites for pin fixation. The posterior tibial artery and tibial nerve pass posteromedially behind the medial malleolus in the distal quarter of the leg. Meanwhile, the sural nerve travels subcutaneously at the distal third of the leg proceeding along the lateral margin of the peroneal tendon and then posterior to the lateral malleolus^26–29^. Given their locations, these structures are at risk during the placement of wires for circular external fixation^30^. Several studies have found safe corridors for the insertion of wires around the ankle joint for circular external fixation^30–32^. However, we are not aware of any study in which the investigators attempted to delineate the safe corridor for the extra-articular calcaneo-tibial pin fixation to avoid neurovascular injury.

A cadaveric study reported on the retrograde calcaneo-tibial fixation of unstable ankle fractures. However, it focused on the efficiency and accuracy of a targeting device developed for the fixation and not on its safety and prevention of neurovascular injury^17^. Another study established an optimal trajectory for calcaneo-tibial K-wire fixation, but it only utilized simple radiographs and focused on the entry angle of the wire to avoid penetrating the ankle, subtalar, or distal tibiofibular joints. The mean entry angle to the plantar plane was 59.4° in the lateral radiograph, while it was 18.4° to the distal tibial articular surface in the mortise radiograph. However, the study did not consider the possibility of neurovascular injury. To ensure a stable fixation, the medial boundary was set at the medial end of the anterior distal tibial articular surface, which had the potential risk of neurovascular injury in our study. In our current study, the distance between the posterior tibial neurovascular structures and the trajectory of the pin from the center of the calcaneal tuberosity to the medial end of the anterior distal tibial articular surface was 5.3 ± 2.2 (range 0.5 to 12.4) mm. The distance became shorter at the proximal area, indicating that it may be risky to aim the pin at the medial end of the anterior distal tibial articular surface on the C-arm fluoroscopic mortise ankle radiograph and proximally to the distal tip of the posterior malleolus on the lateral radiograph. However, aiming the pin lateral to the midpoint of the anterior distal tibial articular surface seemed like a safer option. The mean distance between the posterior tibial neurovascular structures and the trajectory of the pin from the center of the calcaneus to the midpoint of the anterior distal tibial articular surface was 12.8 ± 2.6 (range 6.3 to 20.8) mm. There were no cases wherein neurovascular structures were identified lateral to this trajectory. The mean distance between the sural nerve and the lateral border of the low-risk zone, located from the center of the calcaneal tuberosity to the lateral end of the posterior malleolus at the joint level, was 15.0 ± 2.5 (range 9.1 to 21.1) mm. Similarly, no neurovascular structures were identified medial to this border, indicating that it might be a safe route of insertion. In the lateral radiograph, inserting the pin within 1 cm proximal of the distal tip of the posterior malleolus may be a safe option.

There were several limitations to this study. The distance of the neurovascular structures to the pin was not measured when it had been inserted but was measured on the MRI with an imaginary trajectory and the low-risk zone of the pin on the premise that the ankle is anatomically reduced. The result may be different in the real situation, where the soft tissues are swollen and injured and the ankle is subluxated. However, taking an MRI of the ankle with a metal pin inserted can produce significant artifacts and distorted images. Additionally, the distance was measured on the 2-dimensional image slices but not on the 3-dimensional reconstructed images. A future study with fresh cadavers may confirm the present findings. Another limitation is that the 50 ankles measured in this study may not be enough to confirm the procedure’s safety. Sample size calculation and power analysis may not be appropriate for a non-comparative study without any group allocation. The sample size of 50 ankles was selected based on a previous MRI study that assessed the safe direction of instruments during posterior ankle arthroscopy^21^. The said study included 40 ankle MRIs and measured the distance between the instruments and the posterior neurovascular structures, which is similar to our present study. However, our MRI study included more ankles than this study. Furthermore, to the best of our knowledge, our study included the largest number of ankles to measure the safe distance between the neurovascular structures of the posterior ankle and the instrument. However, there is no clear cut-off distance between the neurovascular structures and the instrument that may be considered safe. In an MRI study, the mean distance between the instrument for posterior arthroscopy and the tibial neurovascular structures and sural nerve was 3.5 and 2.8 mm, respectively. The authors concluded that arthroscopic instruments can be safely used without causing injury to the neurovascular structures^33^. In our study, the distance between the tibial neurovascular structures and the medial border of the low-risk zone was 12.8 mm, while the distance between the sural nerve and the lateral border of the low-risk zone was 15.0 mm. These were farther than the distance presented in the previous MRI study^33^. Moreover, when a pin is inserted within the low-risk zone, the distance between the neurovascular structures and the pin will be even farther compared to the distance between the neurovascular structures and the borders of the low-risk zone. This technique has been clinically applied in nine patients with acute ankle fractures^19^, and none sustained iatrogenic neurovascular injury. In the study, the pin was retained for an average duration of 5.3 weeks (range 2 to 8 weeks), and none of the cases developed a pin-tract infection or pin breakage^19^. However, one patient lost reduction due to noncompliance with weightbearing restrictions. Another limitation is that the current study focused on the safety of the procedure to avoid neurovascular injury but not on mechanical stability. It is evident that a single pin will be less stable compared to a traditional external fixator with a delta frame construct or circular rings. Therefore, this technique is recommended for an unstable ankle or subtalar joint in critical polytrauma patients when time is an essential component of damage control or when the ankle joint is too unstable for a splint or cast immobilization alone and traditional external fixation techniques may not be required or are not cost-effective^19^. We believe that it may be optimal to aim the pin from the center of the calcaneal tuberosity to the center of the distal tibia to ensure stability.

## Conclusions

A MRI analysis demonstrated that an unstable ankle or subtalar joint can be temporarily fixated with an extra-articular calcaneo-tibial pin at a defined zone with a low neurovascular injury risk.

Take home messages.

- To lower neurovascular injury risk during an extra-articular calcaneo-tibial pin fixation, the pin should be inserted from the calcaneal tuberosity center to the distal tip of the posterior malleolus within the low-risk zone.
- A line from the calcaneal tuberosity center to the lateral end of the posterior malleolus at the ankle joint level defines the lateral border of the low-risk zone, medial to which the pin should stay in order to not injure the sural nerve.
- A line from the calcaneal tuberosity center to the midpoint of the anterior distal tibial articular surface at the joint level defines the medial border of the low-risk zone, lateral to which the pin should stay in order to not injure the tibial neurovascular structures.

## Data Availability

The datasets used and analyzed during the current study are available from the corresponding author on reasonable request.
